# Book Notice

**Published:** 1888-06

**Authors:** 


### Book Notice.

We have just received from the Publication Office of the Welsh Dental Co., of Philadelphia, "*The Student's Manual and Hand-Bood for the Dental Laboratory*," by L. P. Haskell of the Dental Department of the North-Western University. This is a little work in which is presented the very essence, in a practical aspect, of Mechanical and Prosthetic Dentistry. It presents clearly and distinctly, though quite briefly, every material point in regard to the laboratory with its complete outfit, together with all the materials required for preparing every kind of artificial substitute. It is very concise and definite in regard to modes and methods of procedure from the beginning to the end, giving that which is necessary, and nothing more. In it is presented very concisely the relative value of the different styles of artificial dentures. There is perhaps no one in the country better fitted for the preparation of such a work than Dr. Haskell. He brings to bear in its preparation the experience, exclusively in this line, of more than forty years. Dr. Haskell has been not only a most indefatigable worker, but he has been a close observer and a thorough experimenter as well, and we may safely say that no one has brought more out of this field of investigation than Dr. Haskell. This is one of the works that we feel safe in unqualifiedly recommending to every dental student and every practitioner as well. It does not supercede nor take the place of larger works already in use, but it certainly fills a niche that has not before been occupied. It can be obtained through any bookseller, and of the dental depots.

